# Alcohol Use Disorder and Dementia: A Review

**DOI:** 10.35946/arcr.v44.1.03

**Published:** 2024-05-23

**Authors:** Natalie M. Zahr

**Affiliations:** Department of Psychiatry and Behavioral Sciences, Stanford University School of Medicine, Stanford, California. Center for Health Sciences, SRI International, Menlo Park, California

**Keywords:** alcohol, aging, Alzheimer disease, neuropsychology, neuropathology, magnetic resonance imaging, positron-emission tomography, rodent

## Abstract

**PURPOSE:**

By 2040, 21.6% of Americans will be over age 65, and the population of those older than age 85 is estimated to reach 14.4 million. Although not causative, older age is a risk factor for dementia: every 5 years beyond age 65, the risk doubles; approximately one-third of those older than age 85 are diagnosed with dementia. As current alcohol consumption among older adults is significantly higher compared to previous generations, a pressing question is whether drinking alcohol increases the risk for Alzheimer’s disease or other forms of dementia.

**SEARCH METHODS:**

Databases explored included PubMed, Web of Science, and ScienceDirect. To accomplish this narrative review on the effects of alcohol consumption on dementia risk, the literature covered included clinical diagnoses, epidemiology, neuropsychology, postmortem pathology, neuroimaging and other biomarkers, and translational studies. Searches conducted between January 12 and August 1, 2023, included the following terms and combinations: “aging,” “alcoholism,” “alcohol use disorder (AUD),” “brain,” “CNS,” “dementia,” “Wernicke,” “Korsakoff,” “Alzheimer,” “vascular,” “frontotemporal,” “Lewy body,” “clinical,” “diagnosis,” “epidemiology,” “pathology,” “autopsy,” “postmortem,” “histology,” “cognitive,” “motor,” “neuropsychological,” “magnetic resonance,” “imaging,” “PET,” “ligand,” “degeneration,” “atrophy,” “translational,” “rodent,” “rat,” “mouse,” “model,” “amyloid,” “neurofibrillary tangles,” “α-synuclein,” or “presenilin.” When relevant, “species” (i.e., “humans” or “other animals”) was selected as an additional filter. Review articles were avoided when possible.

**SEARCH RESULTS:**

The two terms “alcoholism” and “aging” retrieved about 1,350 papers; adding phrases—for example, “postmortem” or “magnetic resonance”—limited the number to fewer than 100 papers. Using the traditional term, “alcoholism” with “dementia” resulted in 876 citations, but using the currently accepted term “alcohol use disorder (AUD)” with “dementia” produced only 87 papers. Similarly, whereas the terms “Alzheimer’s” and “alcoholism” yielded 318 results, “Alzheimer’s” and “alcohol use disorder (AUD)” returned only 40 citations. As pertinent postmortem pathology papers were published in the 1950s and recent animal models of Alzheimer’s disease were created in the early 2000s, articles referenced span the years 1957 to 2024. In total, more than 5,000 articles were considered; about 400 are herein referenced.

**DISCUSSION AND CONCLUSIONS:**

Chronic alcohol misuse accelerates brain aging and contributes to cognitive impairments, including those in the mnemonic domain. The consensus among studies from multiple disciplines, however, is that alcohol misuse can increase the risk for dementia, but not necessarily Alzheimer’s disease. Key issues to consider include the reversibility of brain damage following abstinence from chronic alcohol misuse compared to the degenerative and progressive course of Alzheimer’s disease, and the characteristic presence of protein inclusions in the brains of people with Alzheimer’s disease, which are absent in the brains of those with AUD.

In 2020, an estimated 17% of the U.S. population was older than age 65; this proportion is projected to rise to about 23% by 2060.^1^, ^2^ This prompts an urgent need for identifying potential and modifiable risk factors contributing to health decline.^3^, ^4^ After tobacco, alcohol is the most misused substance in the United States and abroad.^5^ Even prior to the coronavirus disease 2019 (COVID-19) pandemic, which contributed to increased drinking rates, alcohol consumption was notably accelerating in several demographic categories, including in men and women older than age 65.^6^–^8^ Consuming alcohol in harmful patterns—such as binge drinking (five or more drinks in men, or four or more drinks in women, in about 2 hours; where a drink is equivalent to 12 oz beer, 5 oz wine, or 1.5 oz distilled spirits)—occurs in more than 25% of older Americans;^5^, ^9^ annual growth trends in alcohol misuse are reported to be 2.4% in older men and 1.6% in older women.^10^

Although not causative, older age is a risk factor for dementia: Every 5 years beyond age 65, the risk doubles;^11^ and approximately one-third of people over age 85 are diagnosed with dementia.^12^, ^13^ Emerging data support alcohol misuse as a risk factor for dementia.^14^ This review considers the literature to determine whether chronic alcohol misuse increases the risks for (1) alcohol-related dementias, including Wernicke-Korsakoff syndrome (WKS); (2) Alzheimer’s disease; or (3) other forms of dementia (i.e., vascular, frontotemporal, or Lewy body dementia).

## Search Methods and Results

Table 1 presents details regarding the literature searches conducted in preparation for this review. For each section in this article, search terms initially included a combination encompassing alcohol use (e.g., alcohol consumption, alcoholism, binge alcohol, alcohol abuse, alcohol use disorder) and cognitive impairment (e.g., dementia, WKS, Alzheimer’s disease), which were then narrowed to the relevant topic (e.g., clinical diagnoses, epidemiology, neuropsychology). Several search terms describing alcohol use were used as the more traditional term “alcoholism” resulted in far more citation results than the currently accepted term, “alcohol use disorder (AUD).” For example, the combination of the traditional term “alcoholism” with “dementia” resulted in 876 citations, but using the currently accepted term “alcohol use disorder (AUD)” with “dementia” produced only 87 papers. Similarly, whereas the terms “Alzheimer’s” and “alcoholism” yielded 318 results, “Alzheimer’s” and “alcohol use disorder (AUD)” returned only 40 citations. The searches also considered subtypes of dementia in addition to Alzheimer’s disease, such as alcohol-related WKS and vascular, frontotemporal, and Lewy body dementias. Searches regarding animal models (i.e., rat, mouse) were narrowed by pathological terms or relevant mechanisms (e.g., amyloid, neurofibrillary tangles, presenilin).

The two terms “alcoholism” and “aging” retrieved about 1,350 papers; adding phrases (for example, “postmortem” or “magnetic resonance”) limited the number to fewer than 100 papers. As pertinent postmortem pathology papers were published in the 1950s and recent animal models of Alzheimer’s disease were created in the early 2000s, articles referenced span the years 1957 to 2024. In total, more than 5,000 articles were considered; approximately 400 are referenced herein (i.e., only articles directly related to search terms were included).

## Results of the Reviewed Studies

### Human Studies

#### Clinical diagnoses

Diagnoses of psychiatric illnesses typically rely on use of one of two manuals: the *International Classification of Disease* (*ICD*) first published in 1984 by the World Health Organization (WHO; 11th edition [*ICD-11*] implemented in 2022); or the *Diagnostic and Statistical Manual of Mental Disorders* (*DSM*) first printed in 1952 by the American Psychiatric Association (fifth edition [*DSM-5*] released in 2013). *ICD* codes are commonly used by primary care physicians, whereas *DSM* codes are more often used by psychiatrists and psychologists. Complicating consistent diagnoses is the evolution over time of concepts underlying clinical diagnoses of alcohol misuse or dementias. Thus, publications have considered diagnosis rates by comparing criteria in *ICD* to *DSM*,^15^–^17^ *ICD* versions,^18^, ^19^ *DSM-IV* to *DSM-5* AUD,^20^–^24^ *ICD* AUD,^25^ *ICD* neurocognitive disorders,^26^ *DSM* neurocognitive disorders;^27^ bias in AUD^28^, ^29^ and dementia^30^–^32^ diagnoses has also been reviewed.

The diagnosis of an alcohol problem is best made by review of medical histories and interviews with patients. Laboratory tests have low sensitivity, and physical examinations are generally helpful only after the repercussions of alcohol misuse are apparent.^33^–^35^ Consequently, *ICD* diagnoses of AUD in primary care settings typically depend on the presence of health-related conditions, including alcohol-related mental health diagnoses, alcohol-related physical health diagnoses, or evidence for medication prescribed to treat alcohol-related problems.^36^ AUD diagnosed using *DSM-5* requires the patient to meet two of 11 criteria; however, specialists—including psychiatrists, psychologists, social workers, and licensed counselors—use *DSM* criteria for diagnosis with questionable consistency.^24^ Despite extensive public health efforts by the National Institute on Alcohol Abuse and Alcoholism, the Centers for Disease Control and Prevention, and the U.S. Preventive Services Task Force, current estimates are that fewer than 50% of people who visit primary care providers for alcohol-related issues are asked about the problem. Alcohol screening with validated questionnaires—i.e., the 10-question Alcohol Use Disorders Identification Test (AUDIT), the 3-question AUDIT-C on consumption, or the 4-question CAGE (Cut down, Annoyed, Guilty, Eye opener)—occurs in only about 2.5% of primary care visits in the United States.^37^–^39^ The Substance Abuse and Mental Health Services Administration (SAMHSA) is another source of alcohol use data based on self-report.^40^ As with *ICD* and *DSM* diagnoses, recognized limitations of SAMHSA data include frequent methodological changes (e.g., definitions of alcohol misuse), which hamper longitudinal comparisons.^40^ Irrespective of criteria used (i.e., *ICD, DSM*, self-report), AUD is underdiagnosed.^37^,^41^,^42^ Henceforth in this review, “AUD” refers to diagnoses made via any version of *ICD* or *DSM* criteria; otherwise, levels and frequency of alcohol consumption are indicated.

“Dementia” is an umbrella term for a decline in mental (i.e., cognitive, intellectual) functioning that interferes with daily life but does not disturb consciousness or perception. More than 100 subtypes of dementia have been recognized, including proteinopathy (e.g., Alzheimer’s, frontotemporal, Lewy body dementia), vascular (i.e., related to blood vessels), and toxic/metabolic (e.g., alcohol-related, WKS) dementias.^43^, ^44^ *ICD* added the code for Alzheimer’s disease in 1975, and *DSM* added the diagnosis in 1983. Both *ICD-11* and *DSM-5* use the term “neurocognitive impairment” to encompass many types of dementia diagnoses. Diagnosing dementia is difficult owing to its insidious onset as well as the range and diversity of symptoms that can resemble normal aging.^45^, ^46^ Indeed, differential diagnoses are imprecise^47^, ^48^ as the clinical signs and symptoms of the many dementias are essentially the same;^49^, ^50^ criteria and nomenclature for dementia subtypes remain imperfect;^51^–^53^ and selective and specific in vivo biomarkers are still in development.^54^, ^55^ Further, as formal dementia differential diagnoses with currently accepted criteria are resource-intensive, up to 85% of dementia diagnoses are made by non-specialist, primary care clinicians.^56^

#### Epidemiological findings

Patients who develop Alzheimer’s disease may initially present with mild cognitive impairment (MCI), defined as a measurable age-accelerated decline in cognition.^57^ Among patients with documented MCI, one-third progress to a diagnosis of Alzheimer’s disease,^58^ which requires the presence of autopsy-detected neuritic plaques and neurofibrillary tangles.^49^,^57^,^59^ Alzheimer’s disease is frequently diagnosed (50% to 75% of dementia cases), but the diagnosis is rarely validated with imaging (i.e., positron emission tomography [PET]) or postmortem examination.^60^–^63^ When autopsies are conducted, between 12% and 23% of patients diagnosed antemortem with Alzheimer’s disease do not show defining neuropathology, suggesting that current prevalence estimates of Alzheimer’s disease are high.^64^, ^65^ Vascular dementia, the second most diagnosed subtype (up to 20% of cases), often coexists with and is incorrectly diagnosed as Alzheimer’s disease.^66^, ^67^ The remaining dementias are typically categorized as Lewy body, frontotemporal, or alcohol-related.^68^

Compared with other types, alcohol-related dementia tends to have an early onset (i.e., ages 45 to 64) and slow progress.^69^–^71^ In addition to alcohol-related dementia, thiamine deficiency (i.e., Wernicke’s encephalopathy) can occur in settings of high alcohol consumption and in malnutrition due to other causes (e.g., parenteral feeding, bariatric surgery, severe pregnancy-related vomiting).^72^, ^73^ The acute nutritional deficiency is reversible if adequately treated but can otherwise advance to WKS characterized by severe, persistent, cognitive impairment predominantly affecting memory.^74^ In contrast to Alzheimer’s disease, alcohol-related dementia and WKS are more commonly diagnosed in men than women^75^–^77^ and are less likely to be identified as such for several reasons, including underreporting of the extent of alcohol consumption, diagnosis perception bias, and a lack of standardized measures of thiamine.^78^, ^79^

Epidemiological studies support alcohol misuse and AUD as a risk factor for all types of dementia (i.e., collapsed across subtypes). For example, a study in France using *ICD-10* codes to define AUD (codes F10.1–F10.9, Z50.2, F10.20–F10.23) and dementia (codes F00–F03, F05.1, F1x.73, G30, G31, I67.3, R54) found that AUD was a major risk factor for all types, but especially early-onset dementia (before age 65).^77^ A Danish cohort comparing people with *ICD-10*–diagnosed alcohol dependence (code F10.2) and dementia (codes F00–F03, G30) with controls matched on sex, date of birth, and municipality reported twice the hazard ratio for dementia among men and women with alcohol dependence.^80^ A U.S. study of more than 4,000 women veterans over age 55 that used *ICD-9* codes to define AUD (codes 305.00, 305.01, 303.00, 303.01, 303.02, 303.90, 303.91, 303.92) and dementia (i.e., a comprehensive *ICD-9* code list provided by the Veterans Health Administration)^81^ determined that dementia developed in 1.1% of women without AUD and in 3.7% of women with AUD.^60^ The United Kingdom Whitehall II study—using alcohol consumption patterns derived from questionnaires and *ICD-10*–defined dementia (codes F00–F03, F05.1, G30, G31)—demonstrated that, compared with people who drank moderately (i.e., 1 to 14 alcohol units/week), those who drank heavily (i.e., more than 14 alcohol units/week) had increased risk for developing *ICD-10* dementia.^82^ Similarly, an analysis of seven cohorts from the United Kingdom, France, Sweden, and Finland, using self-reported alcohol consumption metrics and *ICD-10* dementia (codes F00–F03, G30, G31, I20–I25, I61, I63–I66, I67.2, I67.3, I67.4, I67.8, I69.3), found that relative to people who drank moderately (i.e., 1 to 14 drinks/week), those who drank heavily (i.e., more than 14 drinks/week) had a 1.2-fold greater risk of developing dementia; and noted associations between high alcohol consumption and early onset dementia.^83^

With respect to the effects of alcohol misuse and AUD on subtypes of dementia, findings are equivocal. A U.S.-based study using data from commercially insured and Medicare Advantage beneficiaries suggested that AUD (*ICD-9* codes 291*, 303*, 305.0*, 357.5, 425.5, 535.3, 571.0, 571.1, 571.2, 571.3; *ICD-10* codes F10*, G31.2, G62.1, G72.1, I42.6, K29.2, K70*, K85.2, K86.0, Q86.0) specifically increased the risk for Alzheimer’s disease (*ICD-9* code 331.0; *ICD-10* codes F00*, G30*).^84^ A study using “driving under the influence” as a proxy for alcohol addiction reported that it was associated with an earlier “Alzheimer’s disease” diagnosis; however, the *ICD-9* codes used in this study (i.e., 290.0–290.3, 290.8–290.9, 331.0) were not specific for Alzheimer’s dementia.^85^ A study using criteria-based diagnoses of dementia and chart-confirmed alcohol misuse (defined as “alcohol consumption that negatively impacts work or social life or leads to legal ramifications”) demonstrated that alcohol misuse was a frequent presenting symptom of frontotemporal but not Alzheimer’s dementia.^86^ Other studies yielded inconclusive results regarding the relationship between alcohol consumption and frontotemporal dementia.^87^, ^88^ Moderate to heavy alcohol consumption (i.e., ≥ 7 drinks/week for women, ≥ 14 drinks/week for men) increased the risk for all types of stroke (i.e., ischemic and hemorrhagic stroke) and may thus be a risk factor for vascular dementia,^89^–^91^ but results are inconsistent.^92^, ^93^

In summary, alcohol misuse and AUD increase risk for all types of dementia. Assuming that 20% of AUD goes unrecognized and 20% of dementias are incorrectly classified as Alzheimer’s disease, one might speculate that a significant proportion of dementia misclassification includes alcohol-related dementia. Reports that AUD specifically increases Alzheimer’s disease likely overestimate the relationship.^94^–^96^

#### Neuropsychological profiles

A constellation of executive cognitive functions—including working memory, set shifting (i.e., the ability to unconsciously shift attention between tasks), problem-solving, and attention—are especially vulnerable to the effects of advancing age.^97^–^99^ The neuropsychological profile of AUD uncomplicated by neurological confounders (e.g., WKS, hepatic encephalopathy) also includes deficits in executive functions.^100^–^102^ Additionally, people with uncomplicated AUD show impairments in episodic memory (i.e., the ability to learn, store, and retrieve information about unique personal experiences including time and place),^103^ visuospatial processing (i.e., the ability to perceive, analyze, and manipulate visual patterns and images, such as copying complex figures or orienting three-dimensional objects),^104^, ^105^ social cognition (i.e., the ability to interpret social information and behave appropriately),^106^, ^107^ and gait and balance.^108^

Features of WKS are persistent inability to remember new information (i.e., anterograde amnesia) and occasional confabulation.^74^, ^109^ Compared with non-alcohol-related WKS, the neuropsychological profile of alcohol-related WKS is broader and commonly includes executive dysfunction.^110^–^113^

Meta-analyses suggest that immediate and delayed memory tests (e.g., word-list recall) have high diagnostic accuracy in differentiating people with Alzheimer’s disease from individuals without the disease but poorly discriminate those with and without MCI.^114^, ^115^ Among available tools, the Montreal Cognitive Assessment (score ≤ 24), the Mini-Mental State Examination (MMSE, score ≤ 26), and the Dementia Rating Scale (score ≤ 124) appear to be efficient at discriminating MCI from aging without cognitive impairment.^116^, ^117^

Refined neuropsychological data can help distinguish dementia subtypes. For example, people with Alzheimer’s disease have more severe deficits in working and delayed memory than do those with WKS.^118^–^120^ In people with AUD or Alzheimer’s disease, the degree of impairment in verbal fluency, working memory, and frontal functions can be similar; memory problems, however, are more pronounced in Alzheimer’s disease relative to AUD.^121^ Similarly, although individuals with alcohol-related dementia or vascular dementia can show executive control deficits, they have less severe memory impairments than observed in those with Alzheimer’s disease.^122^ Further, patients with alcohol-related dementia demonstrate stabilization of functional impairment with abstinence, whereas those with Alzheimer’s disease or vascular dementia show a progressive decline in cognitive functions.^123^ Indeed, in a longitudinal study, people with alcohol-related dementia with monitored abstinence showed improved performance on executive functioning tests, whereas people with Alzheimer’s disease performed worse on memory tests over the same time spans.^124^ The amount of alcohol consumed was unrelated to cognitive performance in patients with *DSM-III*–defined “primary degenerative dementia.”^125^ In a more recent study of people diagnosed with MCI (*ICD-10* code F067) and evaluated by structured interview for alcohol use—i.e., low (less than 1 drink/week), moderate (1 to 14 drinks/week for men and 1 to 9 drinks/week for women), or heavy (more than 14 drinks/week for men and more than 9 drinks/week for women)—levels of alcohol consumed had no effect on MMSE scores; however, MMSE scores are notoriously insensitive to AUD-related cognitive decline.^126^, ^127^

In summary, neuropsychological profiles differ between people with healthy aging, AUD, WKS, Alzheimer’s disease, and other subtypes of dementias. AUD adds a burden to aging in the executive domain. Although AUD, WKS, and Alzheimer’s disease all affect memory processes, the effects of Alzheimer’s disease on mnemonic functions are greater than those observed in AUD and WKS.

#### Postmortem neuropathology

Normal aging decreases the brain’s viability and increases its vulnerability to damage,^128^, ^129^ but neuronal loss is not a salient feature.^130^–^132^ Instead, careful stereological studies have concluded that age-related changes in the central nervous system (CNS) in the cognitively intact, aging brain include alterations to neuron extensions (e.g., retraction of dendritic arbors and synapses);^133^, ^134^ deterioration of non-neuronal cells (e.g., oligodendrocytes, astrocytes, microglia);^135^–^138^ and biochemical and molecular changes (e.g., reduced efficacy of neurotransmitters).^139^–^142^ These effects of aging in the healthy brain differ from those seen with pathological aging due to neurological conditions such as Alzheimer’s disease.^143^, ^144^ A cardinal pathological feature of Alzheimer’s CNS tissue, which has been known for more than 100 years, is the progressive accumulation of insoluble fibrous materials, including extracellular plaques of beta-amyloid (A-beta), which has two major isoforms (A-beta-42 and A-beta-40), and intraneuronal neurofibrillary tangles composed of the microtubule-binding protein tau.^145^–^147^ The cause, effect, and reciprocity of A-beta and tau accumulation with neurodegeneration and symptoms of dementia are the subject of ongoing debates.^49^,^57^,^59^,^148^ Nevertheless, substantiation of an Alzheimer’s diagnosis continues to require postmortem identification of these characteristic protein inclusions in regions including the entorhinal cortex and hippocampus, where they contribute to severe neuronal loss and salient impairment in memory consolidation of newly experienced events.^149^, ^150^ Neuropathological observations further suggest that neuronal loss in a specific area of the hippocampus (i.e., subfield CA1) may be a specific marker for Alzheimer’s disease.^151^–^153^

Other proteinopathies also present with neuropathological inclusions. Lewy body dementia is characterized by presence of protein aggregates (Lewy bodies) containing alpha-synuclein,^154^ whereas frontotemporal dementia is associated with tau and TDP-43 (transactive DNA binding protein of about 43 kDa) pathology in at least 50% of cases.^155^–^157^ In vascular dementia, gross examination of the brain may reveal overt lesions, microinfarcts, or damage to blood vessels, and microscopic evaluation may detect accumulation of lipids or blood clots.^158^, ^159^ Other postmortem signs of vascular disease include white matter atrophy and calcification of arteries.^43^,^160^,^161^

A coordinated cross-sectional analysis of six community-based autopsy cohorts in the United States and the United Kingdom highlighted the complexity of the brain pathologies that underlie dementia. The analysis assessed 12 dementia-related pathologies in brains of those age 80 and older, including vascular pathologies (arteriolosclerosis, atherosclerosis, microinfarcts, lacunes, and cerebral amyloid angiopathy); Alzheimer’s disease-related pathologies (Braak neurofibrillary tangle stage, Consortium to Establish a Registry for Alzheimer’s Disease [CERAD] diffuse plaque score, CERAD neuritic plaque score, and hippocampal sclerosis); Lewy body dementia pathology; and TDP-43 pathology. Of the overall sample, which generally included more women than men, 40% had vascular-related pathology, 70% had Alzheimer’s disease-related pathology, and 68% of the cohort had pathology co-occurrence.^162^ Smaller studies similarly reported a high frequency of coincident neuropathology.^163^, ^164^

WKS does not have clear neuropathological markers. Careful stereological approaches, however, have demonstrated neuronal loss in medial thalamus, mammillary bodies, pons, medulla, and anterior-superior vermis of the cerebellum.^165^, ^166^ A series of neuropathological analyses compared the effects of alcohol per se to distinct neurological conditions associated with chronic alcohol consumption, including WKS, hepatocerebral degeneration, Marchiafava Bignami disease, and central pontine myelinolysis. The studies concluded that alcohol as such does not contribute to a progressive or irreversible pathology.^118^,^167^–^170^ Instead, quantitative histological analyses of individuals with uncomplicated AUD often use the term “alcohol-related brain damage” to refer to the plastic CNS changes associated with chronic alcohol use as discrete from neurodegenerative disease.^171^, ^172^ Tissue loss occurring mainly in the frontal lobes and cerebellum of the brain in people with AUD is not associated with neuronal death.^173^–^177^ Indeed, no changes in neuron numbers have been documented in brain tissue (e.g., hippocampus, basal ganglia, serotonergic raphe nuclei, cholinergic basal forebrain) from people with AUD without liver pathology, nutritional deficiencies, or other complications.^177^–^182^ AUD-related neuropathological changes are instead largely accounted for by retraction of dendritic arbors and shrinkage of white matter.^173^,^174^,^183^–^188^

Alzheimer’s disease-related protein markers (i.e., A-beta, tau) are not affected by alcohol consumption. For example, A-beta plaques were not increased in the brains of people who drank heavily (more than 6 drinks per day for at least 10 years).^189^, ^190^ Further, men who drank moderately (not more than 4 drinks/day or 14 drinks/week) showed less neurofibrillary tangle pathology compared with men who drank never or heavily.^191^ In a study of individuals with thiamine deficiency who who drank alcohol chronically, neurofibrillary pathology was found in the nucleus basalis (which is affected in WKS) but not in any other brain region.^192^ Further, heavy alcohol consumption (i.e., daily, socially disabling alcohol use, and continued drinking despite indisputable health-related or social damage) showed no statistically significant influence on the extent of alpha-synuclein pathology or incidence of total infarcts;^193^ however, very heavy alcohol consumption (more than 32 drinks/week) may increase hemorrhagic stroke.^194^

In summary, evidence from postmortem histological analyses indicates that healthy CNS aging and AUD are not associated with significant neuronal loss, whereas Alzheimer’s disease and WKS show regionally specific neurodegeneration. Based on postmortem evaluations, uncomplicated AUD does not contribute to archetypal Alzheimer’s disease pathology characterized by the presence of protein inclusions.

#### Neuroimaging biomarkers

An advantage of in vivo neuroimaging over postmortem study is the ability to track individuals longitudinally, which permits evaluation of causative factors in CNS volume changes and the consequences of behavioral modifications (e.g., cessation of alcohol drinking). Cross-sectional and longitudinal magnetic resonance imaging (MRI) studies in adults have provided consistent evidence for systematic, age-related volume increases in spaces filled with cerebrospinal fluid (CSF)—i.e., sulci, fissures, and ventricles—that occur at the expense of gray matter and may accelerate with older age.^195^–^201^ Brain gray matter structures exhibit differential patterns of aging, with convergent longitudinal data indicating an excessive vulnerability of prefrontal cortex.^202^–^206^ Age-related volume deficits in thalamus and cerebellum occur at a slower rate than declines in cortical gray matter.^207^–^211^ Gross white matter volume remains relatively stable across adulthood;^201^,^212^–^214^ however, appropriate imaging modalities (e.g., fluid-attenuated inversion recovery, diffusion tensor imaging) demonstrate more hyperintense inclusions (i.e., white matter hyperintensities [WMH]),^215^–^218^ and microstructural compromise in older relative to younger individuals.^219^–^222^

Cross-sectional neuroimaging reports support AUD-related volume shrinkage in specific brain structures, including frontal, temporal, and parietal cortices; diencephalon; brain stem; and cerebellum.^223^–^229^ In contrast to results of postmortem analyses of neuronal numbers, neuroimaging studies describe significant volume deficits in people with AUD, relative to healthy controls, in hippocampus and basal ganglia (i.e., caudate, putamen, nucleus accumbens) that may be accounted for by white matter compromise.^224^,^230^–^235^ Longitudinal studies that compare individuals with older age at AUD onset and relatively less lifetime alcohol use with individuals with younger age at AUD onset further support accelerated brain aging in frontal cortical volumes due to age–alcohol interactions and not just attributable to more years of alcohol misuse.^227^,^236^–^239^ Other longitudinal studies show that alcohol abstinence is associated with brain integrity improvement (i.e., volume recovery), whereas relapse precipitates further volume shrinkage.^240^–^244^ Individuals with AUD who relapse show continuing volume decline compared with those who achieve abstinence,^225^,^241^,^245^,^246^ but even reduced drinking without achieving or maintaining complete abstinence can improve brain structure and function.^247^ Similarly, a controlled longitudinal study that assessed individuals with AUD soon after withdrawal and then again after 2 weeks of sobriety suggested resolution of volume deficits specifically in hippocampal subfield CA2+3^248^ (also see Zahr et al., 2019^232^; Lee et al., 2016^249^). This reversibility of volume deficits with abstinence is in stark contrast to the irrevocable progression of Alzheimer’s disease.^250^, ^251^

Acute Wernicke’s encephalopathy also has characteristic changes evident on transverse relaxation time (T2)-weighted images showing bilateral, high signal intensities in the periaqueductal gray, mammillary bodies, thalamus, and hypothalamus.^252^–^254^ Quantitative MRI documents a graded pattern of accruing volume deficits in hippocampus, thalamus, mammillary bodies, cerebellum, and pons as disease severity progresses from AUD to WKS.^230^,^255^,^256^ Mammillary body shrinkage has been suggested as being able to differentiate WKS from Alzheimer’s disease,^257^, ^258^ as have diffusion tensor imaging (DTI) metrics indicating abnormalities in anterior thalamus to hippocampus white matter tracts.^259^

Deviations of hippocampal volume from normal age-related decline have been identified as a sensitive indicator of Alzheimer’s disease pathology.^22^,^234^,^260^,^261^ Indeed, atrophy of entorhinal cortex and hippocampus may distinguish Alzheimer’s disease from healthy aging with up to 90% accuracy;^262^, ^263^ further, the rate and extent of CA1 atrophy may help distinguish Alzheimer’s disease from other forms of dementia.^241^,^264^–^267^ Longitudinal studies suggest that the pattern of gray matter atrophy in people with MCI who are later diagnosed with Alzheimer’s disease mimics the pattern of atrophy observed in Alzheimer’s disease but is less extreme. However, in people with MCI who do not eventually receive an Alzheimer’s disease diagnosis, the pattern of gray matter atrophy is more comparable to that observed in healthy elderly individuals without dementia.^268^–^270^ Similarly, detrimental changes in regional (e.g., fornix, uncinate, cingulum) diffusivity in MCI quantified using DTI are less pronounced than those observed in people with Alzheimer’s disease.^114^,^271^,^272^

A research framework for diagnosing Alzheimer’s disease, released by the National Institute on Aging in 2018, integrated neuroimaging biomarkers A, T, and N. In this framework, A represents A-beta plaques determined by cortical amyloid PET ligand binding (or CSF A-beta-42 levels); T represents fibrillar tau protein, determined by cortical tau PET ligand binding (or CSF phosphorylated tau [p-tau] levels); and N represents neuronal injury or neurodegeneration determined with fluorodeoxyglucose PET hypometabolism or MRI volume (typically hippocampal) atrophy.^273^–^275^ These three markers are used to distinguish among eight dementia profiles: normal, healthy individuals (A−T−N−); people with a condition along the Alzheimer’s disease continuum (A+T−N−; A+T−N+; A+T+N−; A+T+N+); and people with non-Alzheimer’s changes (A−T+N−; A−T+N+; A−T−N+).^57^, ^276^

Vascular dementias (which include at least six subtypes) are identified on MRI by presence of infarcts, small cavities (lacunes), and WMH.^277^–^280^ WMH are considered a neuroimaging feature of cerebral small vessel disease that can increase the risk for stroke and vascular dementia.^281^, ^282^ As they are ubiquitous and heterogeneous, however, a better characterization of the extent, distribution, and cognitive correlates of WMH is necessary.^283^–^285^ In support of a high co-occurrence of Alzheimer’s disease and vascular dementias, a literature review found a strong relationship between presence of amyloid and WMH burden^286^ (also see Eloyan et al., 2023^287^).

Although separate structural neuroimaging studies in people with AUD, WKS, or Alzheimer’s disease report gray matter volume loss in common regions, including hippocampus,^258^,^288^,^289^ a direct comparison among these patient groups demonstrates that hippocampal volume loss in people with AUD relative to Alzheimer’s disease is less severe.^290^ Further, PET markers that characterize Alzheimer’s disease are not elevated in people with AUD. Two PET studies using the Pittsburgh Compound-B ([11C]PiB) ligand found no significant differences in global A-beta loads between people with AUD and healthy control study participants^291^, ^292^ (also see Mendes et al., 2018^293^). Another report found that compared with no drinking, moderate drinking (1 to 13 drinks/week) was associated with lower [11C]PiB-determined A-beta deposition.^294^ In contrast, people with AUD had larger WMH volumes than did healthy controls, suggesting an increased cerebrovascular risk in AUD.^207^, ^292^

In summary, healthy aging is characterized by nonlinear gray matter volume decreases, particularly in frontal regions; slower white matter decline; and a greater incidence, compared to younger brains, of WMH.^227^,^295^–^298^ AUD can amplify the severity and extent of age-related volume decline, especially in frontal regions, but abstinence is associated with significant volume recovery.^246^, ^299^ In vivo diagnosis of Alzheimer’s disease necessitates PET imaging, but available evidence does not support AUD as contributing to Alzheimer’s disease PET markers. In vivo MRI of individuals with Alzheimer’s disease can demonstrate greater than age-corrected hippocampal atrophy, but deviations from age-related changes can be challenging to quantify. Instead, emerging data suggest that hippocampal subfield analyses (e.g., effects on CA1 in Alzheimer’s disease and on CA2+3 in AUD) may help with future differential diagnoses.

#### CSF and blood biomarkers

The National Institute on Aging research framework supports CSF quantification of extracellular A-beta-42 and p-tau for accurate and early diagnosis of Alzheimer’s disease, but optimization and standardization of these measures is in progress.^300^–^302^ CSF A-beta-42 levels are low in people with Alzheimer’s disease compared to unaffected controls and reflect an increase in CNS amyloid plaques.^303^–^305^ Low CSF levels of A-beta-42 also can predict MCI and conversion from MCI to Alzheimer’s disease.^306^, ^307^ As levels of CSF A-beta-42 are also low in Lewy body, vascular, and frontotemporal dementias, however, A-beta isoforms are being explored to help to differentiate dementia subtypes.^308^–^310^ Levels of CSF tau, p-tau, and their epitopes are high in people with Alzheimer’s disease compared to unaffected subjects and may indicate hippocampal atrophy, but levels of these CSF proteins are also high relative to healthy controls in other neurodegenerative diseases.^311^–^313^ Combinations and ratios (e.g., A-beta-42/A-beta-40) of CSF A-beta-42, total tau, and p-tau and their variants are under investigation to improve success of differential diagnoses.^314^, ^315^

Total tau is significantly elevated in people with acute Wernicke’s encephalopathy, but the overall pattern of CSF changes (involving A-beta, total tau, and p-tau) can clearly distinguish acute and chronic WKS from Alzheimer’s disease.^316^ CSF tau and A-beta markers are present in only 11% of AUD patients with cognitive deficits;^317^ conversely, alcohol misuse is rarely observed in those with Alzheimer’s disease biomarkers.^318^ Thus, CSF tau and A-beta markers may be useful in differentiating alcohol-related cognitive disorders from Alzheimer’s disease.^319^

Although neuroimaging and CSF markers approved by the U.S. Food and Drug Administration can aid in detection and diagnosis of Alzheimer’s disease, the clinical implementation of these testing modalities is limited because of their availability, cost, and perceived invasiveness.^320^ Blood-based markers are also in development for earlier, faster, and more accessible diagnoses.^321^ Associations between blood and CSF tau and A-beta and other disease markers, however, and their ability to help with differential diagnoses are not fully established.^322^–^325^

#### Summary of human studies

The consensus among studies from multiple disciplines is that AUD can increase the risk for dementia, but not necessarily the risk of Alzheimer’s disease. A review of clinical and epidemiological data suggests that criteria and nomenclature of dementia subtypes need improvement. Neuropsychological and biological markers that can differentiate dementia subtypes are in progress but currently limited. Whether alcohol misuse contributes to an added burden on pre-existing Alzheimer’s disease remains an open and ongoing research question, which may be approached in animal models. Indeed, basic science strategies that can control alcohol exposure may help clarify controversies, including whether alcohol in the context of genetically induced Alzheimer’s disease pathology changes the extent, distribution, or signaling pathways of relevant biomarkers.

### Translational Studies

#### Rodent models of AUD

In contrast to the human brain, the rat brain increases in weight and length with advancing age and demonstrates continued growth in older (e.g., age 763 days) relative to younger (e.g., age 109 days) rodents.^326^, ^327^ Longitudinal imaging studies that followed animals for up to 19 months confirm accrual of body weight and total brain volume with increasing age in wild-type Wistar rats, alcohol-preferring (P) and non-preferring (NP) strains derived from Wistar rats, and Fischer 344 (F344) rats.^220^,^228^,^328^–^330^ MRI studies further show an aging-related pattern in rats contrary to that observed in humans: Total CSF, gray matter, and white matter volumes continue to increase with older age.^228^, ^331^ These fundamental differences in CNS aging between rodents and humans are critical to model in studies that consider the combined effects of ethanol (EtOH) exposure and Alzheimer’s disease-related pathology.

Several susceptible brain regions have been demonstrated in rodents exposed to high EtOH levels via intragastric,^332^ intraperitoneal (i.p.),^333^, ^334^ or vapor^335^, ^336^ protocols. Immunohistochemical staining procedures highlight degenerative effects of EtOH on corticolimbic circuitry.^337^–^342^ By contrast, unbiased screening approaches that indicate neuronal activity (e.g., glucose utilization, c-Fos expression) but not loss identify different regions affected by EtOH, including thalamus, colliculi, cerebellum, and pons.^343^–^346^ Longitudinal neuroimaging findings consistently report ventricular enlargement in response to binge and chronic EtOH exposure that is reversible upon abstinence.^228^,^330^,^347^–^349^ Indeed, among regions demonstrating reduced volume following EtOH exposure (e.g., retrosplenial and cingulate cortices, dorsal hippocampi, central and ventroposterior thalami, corpus callosum), most show significant recovery with abstinence.^350^, ^351^ Volumes of the colliculi and periaqueductal gray, however, show persistent volume deficits with abstinence.^350^, ^351^ Although the colliculi may be relevant to human AUD, they have rarely been investigated in humans, possibly because of the challenges in visualizing and quantifying colliculi by MRI.^352^

Relatively few papers have explored the effects of EtOH on the aged rodent brain. Following a single i.p. EtOH dose, older (18 months) compared with younger (postnatal days 70 to 72) Sprague Dawley rats showed greater EtOH-induced ataxia (accelerating rotarod, aerial righting reflex) and cognitive impairment (i.e., longer latency to locate submerged platform on the Morris water maze).^353^ However, against expectations, a longitudinal in vivo study of F344 rats exposed to intragastric EtOH for 4 days^354^ showed greater transient tissue volume compromise in young rats (age 4 months) compared to older rats (age 17 months).^331^ By contrast, EtOH administration alters markers of astrocytes and microglia more significantly in older than younger animals. For example, chronic moderate EtOH exposure (daily 2 g/kg, i.p. doses for 45 days) increased glial fibrillary acidic protein (GFAP, an astrocyte protein expression marker) to a greater extent in older (age 19 months) than younger (age 3 months) Wistar rats.^355^ Similarly, a microglial mRNA marker that increased in response to EtOH resolved with abstinence in young but not older C57BL/6J mice^356^ (also see Marsland et al., 2022^357^).

#### Rodent models of Alzheimer’s disease

Several genetically modified (i.e., transgenic) mouse models of Alzheimer’s disease are now available. The first models used various constructs to overexpress amyloid precursor protein (APP), which is processed in the body by enzymes (i.e., beta- and gamma-secretases) to generate soluble amyloid peptide (A-beta) fragments.^358^ Mice with overproduction of total A-beta from APP exhibit extracellular A-beta deposits reminiscent of plaques in human brains as well as cognitive dysfunction.^359^–^361^ However, these animals did not have neurofibrillary tangles or show neuronal loss. Second-generation mutant mice included overexpression of presenilin (PS), a constituent of the gamma-secretase complex that cleaves APP.^362^ PS1 overexpression alone did not induce A-beta pathology;^363^ however, the combined expression of APP and PS1 increased pathogenic A-beta production and deposition, behavioral deficits, and neuronal loss.^364^–^367^ One of these models was the 5XFAD mouse line, which expresses five human APP and PS1 transgenes and results in mice with A-beta pathology, gliosis, synaptic degeneration, neuronal loss, and progressive cognitive deficits as early as 4 months of age.^368^ Despite their aggressive phenotypes, these models also failed to develop neurofibrillary tangles. In efforts to replicate neurofibrillary tangle pathology, a mouse line was created that carried targeted insertions (knock-in mutations) of PS1, APP, and microtubule-associated protein tau (i.e., 3xTg-AD mice).^369^ The 3xTg-AD mouse line is a well-validated animal model that develops rapid, age-dependent, and progressive Alzheimer’s-like neuropathology, including A-beta and tau tangles.^370^–^372^

Although widely used, these models imitate only certain aspects of human Alzheimer’s disease pathology.^373^–^375^ Further, the amyloid peptides generated by mice are distinct from those produced by the human brain.^376^ Such gaps have led to a program initiated by the National Institute on Aging—the Model Organism Development and Evaluation for Late-Onset Alzheimer’s Disease (https://www.model-ad.org)—to fund development of Alzheimer’s disease mouse models that better recapitulate the human disease.

#### Rodent models of AUD and Alzheimer’s disease

Only a few studies have evaluated how EtOH may exacerbate Alzheimer’s-related behavior and brain pathology in wild-type rodents. Compared to unexposed mice, wild-type C57BL/6J mice exposed to EtOH (1 month, free access to water, 10% or 20% EtOH) showed impaired spatial memory and elevated hippocampal p-tau, but no change in total tau.^377^ Similarly, wild-type, male C57BL/6J mice exposed to both EtOH (via liquid diet for 7 weeks at 28% of total calories) and thiamine deficiency demonstrated nonspecific, whole-brain increases in A-beta (both A-beta-42 and A-beta-40 isoforms) protein levels compared to unexposed mice^378^ (also see Zhao et al., 2011^379^). Finally, compared with unexposed animals, Sprague Dawley rats exposed to EtOH (via liquid diet for 5 weeks at about 36% of total calories) showed increased expression of APP and beta-site APP-cleaving enzyme 1 (BACE1, which is critical for A-beta expression) in hippocampus, cerebellum, and striatum.^380^ Of note, nonspecific, elevated levels of A-beta also have been observed in response to other age-related pathologies (e.g., hypertension, diabetes^381^, ^382^), and elevations in p-tau can occur in response to other, particularly anesthetic, psychoactive agents.^383^, ^384^

Findings observed in wild-type animals appear to be exaggerated in transgenic mice. For example, APP/PS1 mice exposed to EtOH (drinking in the dark for 1 month), compared to vehicle-treated APP/PS1 animals, showed greater memory deficits (i.e., Morris water maze performance), higher whole-brain APP and BACE1 levels, and enhanced plaque formation.^385^ Similarly, compared with unexposed mice, APP/PS mice exposed to 10 weeks of moderate EtOH in a two-bottle choice design showed deficits in nest building (but not in an object location memory task), and a higher frequency of A-beta deposition and plaques in hippocampus.^386^ Also in APP/PS transgenic mice, binge EtOH treatment during adolescence (via four i.p. injections per week of 2.5 g/kg EtOH during postnatal days 20 to 60) increased A-beta RNA and protein expression in the hippocampus at ages 6 and 12 months.^387^ In 3xTg-AD mice—the only transgenic model able to produce both A-beta and tau markers—EtOH exposed (via 4-month, free access to water or 25% EtOH), compared with saccharin-exposed (control) 3xTg-AD mice, showed impaired spatial memory on the Morris water maze and upregulated A-beta-42/40, total tau, and p-tau 1 month after EtOH exposure.^388^ Another study showed that EtOH exposure (6 weeks of 4 days/week vaporized EtOH) to 3xTg-AD mice hastened cognitive impairment and increased levels of a different protein marker, alpha-synuclein (relevant to Lewy body dementias).^389^

Recent translational work highlights sex differences in the interaction of EtOH with Alzheimer’s disease-related pathology. EtOH exposure caused greater cognitive impairment in female than male “middle aged” (ages 6 to 9 months) wild-type C57BL/6J mice,^390^ which was associated with an increase in hippocampal amyloid levels.^391^ In mice with abnormal tau deposition (i.e., PS19 model with the T34 tau isoform), 16 weeks of intermittent access to water containing 20% EtOH increased the excitability of the locus coeruleus more in female than male mice.^392^ Finally, 3xTg-AD adolescent and adult mice exposed to EtOH showed EtOH-related increases in total and hyperphosphorylated tau in female mice but not in male mice, which were hypothesized to be related to impaired lysosome function.^393^, ^394^ These recent papers demonstrating EtOH effects in only female transgenic mice^393^, ^394^ acknowledged previous findings that total tau and p-tau were increased in both sexes of 3xTg-AD mice,^388^ but did not comment on the underlying reasons for such discrepancies. Indeed, the relevance of sex-related findings in transgenic rodents to the human condition await a better understanding of the pathological mechanisms underpinning Alzheimer’s disease.

## Conclusions

Limitations of the current narrative review are that it failed to address all nuances of the potential relationship between alcohol misuse and dementia risk. For example, the contributions of a genetic predisposition to Alzheimer’s disease (i.e., presence of the apolipoprotein E epsilon4 allele, the major genetic risk factor) to the various metrics were not considered.^92^, ^395^ Further, an emerging literature showing a relationship between liver pathology—including alcohol-related liver disease—and Alzheimer’s disease was not explored.^396^–^398^

This literature review indicates that chronic alcohol misuse accelerates brain aging and contributes to cognitive impairments, including those in the mnemonic domain also affected in Alzheimer’s disease. The current literature analysis, however, agrees with a 2001 review published in this journal that alcohol misuse does not increase the risk for Alzheimer’s disease per se.^399^ Whether alcohol misuse or AUD increase the risk for alcohol-related or other forms of dementia may be clarified by improvements in neuropsychological tests or biomarkers better able to differentiate dementias in vivo.

## Figures and Tables

**Table 1:** Literature Search Details

**Relation evaluated**	Alcohol consumption and dementia	

**Databases used**	PubMed, Web of Science, and ScienceDirect	

**Literature covered**	Clinical diagnoses, epidemiological findings, neuroimaging, neuropsychological profiles, other biomarkers, postmortem pathology, and translational studies	

**Literature search dates**	January 12, 2023–August 1, 2023	

**Literature search terms**	“aging,” “alcoholism,” “alcohol use disorder (AUD),” “brain,” “CNS,” “dementia,” “Wernicke,” “Korsakoff,” “Alzheimer,” “vascular,” “frontotemporal,” “Lewy body,” “clinical,” “diagnosis,” “epidemiology,” “pathology,” “autopsy,” “postmortem,” “histology,” “cognitive,” “motor,” “neuropsychological,” “magnetic resonance,” “imaging,” “PET,” “ligand,” “degeneration,” “atrophy,” “translational,” “rodent,” “rat,” “mouse,” “model,” “amyloid,” “neurofibrillary tangles,” “α-synuclein,” “presenilin”	

**Additional filters**	Species (i.e., “humans” or “other animals”)	

**Results** *	1,339	“alcoholism” and “aging”
	876	“alcoholism” and “dementia”
	498	“alcohol consumption” and “dementia”
	318	“Alzheimer’s” and “alcoholism”
	231	“Alzheimer’s” and “alcohol consumption”
	87	“alcohol use disorder (AUD)” and “dementia”
	60	“alcoholism” and “aging” and “magnetic resonance”
	40	“Alzheimer’s” and “alcohol use disorder (AUD)”
	31	“alcoholism” and “aging” and “postmortem”

* Source: PubMed, August 14, 2023.
